# A Western Diet Ecological Module Identified from the ‘Humanized’ Mouse Microbiota Predicts Diet in Adults and Formula Feeding in Children

**DOI:** 10.1371/journal.pone.0083689

**Published:** 2013-12-31

**Authors:** Jay Siddharth, Nicholas Holway, Scott J. Parkinson

**Affiliations:** 1 Host Commensal Hub, Developmental and Molecular Pathways, Novartis Institutes for Biomedical Research, Basel, Switzerland; 2 Scientific Computing, NIBR IT, Novartis Institutes Biomedical Research, Basel, Switzerland; Argonne National Laboratory, United States of America

## Abstract

The interplay between diet and the microbiota has been implicated in the growing frequency of chronic diseases associated with the Western lifestyle. However, the complexity and variability of microbial ecology in humans and preclinical models has hampered identification of the molecular mechanisms underlying the association of the microbiota in this context. We sought to address two key questions. Can the microbial ecology of preclinical models predict human populations? And can we identify underlying principles that surpass the plasticity of microbial ecology in humans? To do this, we focused our study on diet; perhaps the most influential factor determining the composition of the gut microbiota. Beginning with a study in ‘humanized’ mice we identified an interactive module of 9 genera allied with Western diet intake. This module was applied to a controlled dietary study in humans. The abundance of the Western ecological module correctly predicted the dietary intake of 19/21 top and 21/21 of the bottom quartile samples inclusive of all 5 Western and ‘low-fat’ diet subjects, respectively. In 98 volunteers the abundance of the Western module correlated appropriately with dietary intake of saturated fatty acids, fat-soluble vitamins and fiber. Furthermore, it correlated with the geographical location and dietary habits of healthy adults from the Western, developing and third world. The module was also coupled to dietary intake in children (and piglets) correlating with formula (vs breast) feeding and associated with a precipitous development of the ecological module in young children. Our study provides a conceptual platform to translate microbial ecology from preclinical models to humans and identifies an ecological network module underlying the association of the gut microbiota with Western dietary habits.

## Introduction

As part of the acceleration of economic globalization in the last quarter of the 20^th^ century came a realization that the ‘Western Lifestyle’ is primarily responsible for the forecasted epidemic in chronic diseases. A combination of inactivity and rapid changes in dietary habits are now recognized as major contributing factors to the pathogenesis of cancer, obesity, diabetes, cardiovascular diseases as well as other chronic inflammatory diseases in the developing world. Recent shifts evident in the developing world towards an ‘energy dense’ diet comprised of animal fat and processed foods alongside reduced complex carbohydrate and dietary fiber intake parallel the predicted dominance of chronic over infectious disease death rates across most of the world [1]. Metabolism of our food is coordinated by the gut microbiota capable of extracting nutrients consumed in the diet [2]. Therefore, there has been growing interest in understanding the relationships between diet, microbes responsible for metabolism and their associated links with chronic diseases. However, comprehension of the molecular mechanisms that link microbial ecology in the context of human health has not kept pace with the publication of technology-driven catalogs of the flora associated with health status [3]–[5].

Two major challenges associated with the translation of exploratory research findings concerning the microbiota can be summarized with two questions. Firstly, can the microbial ecology of preclinical models be used to predict biological traits in human populations? And secondly, can the plasticity of microbial ecology in humans be harnessed to predict specific populations? With these questions in mind, we decided to take an alternative look at two recent papers investigating the impact of dietary intake. One of these studies investigated a humanized mouse model, where gnotobiotic mice were inoculated with human donor feces and subsequently challenged with a controlled diet. Using principle component analysis, the authors concluded that individual variation in microbiota composition is more significant than microbial changes following dietary changes, consistent with the concept of microbial ‘enterotypes’ previously put forward [4], [5]. The ‘enterotype’ concept introduced the perception that the individual composition of the microbiota is relatively stable and resistant to environmental influence. Similar conclusions were put forward by Wu et al. following their analysis of the composition of the microbiota from a human dietary intervention study conducted in a controlled environment [3]. The investigators recruited 10 volunteers who were assigned to either a Western or low fat/high fiber dietary group. In the same report, they also looked at the dietary intake of human volunteers in context with the composition of their microbiota. The authors concluded that while dietary change influenced the microbiota of the individual, the composition of the microbiota was dominated by the individual ‘enterotype.’

The conclusions of these studies pose some practical challenges with respect to the translation of microbiota research to applications of consequence to human health. Of concern for pharmaceutical applications, is if the source dominates the microbial profile, how can we use preclinical models to support drug-discovery efforts or disease monitoring? In addition, if individual ‘enterotypes’ dominate microbial composition, how can the microbiota be used to predict response, monitor outcomes or identify specific drug-resistant or sensitive populations?

We took an alternative view to the ‘enterotype’ approach to specifically address these questions. We considered the composition of the microbiota as a cooperative group of individual bacteria that interact with neighbors to establish an ecological network module (or ‘module’ for short) whereby they can optimally use nutritional intake to their advantage. Our hypothesis was that if these communities could be identified, they should correlate with specific intervention (in this case dietary intake) regardless of the original composition (or ‘enterotype’) of the microbiota. We applied this approach to the outlined published studies and identified an interactive module of 9 genera allied with Western diet in the ‘humanized’ mice. We then applied this module to the controlled dietary study in humans predicting dietary intake in patients. In the same study, the module also correlated with individual dietary intake of 98 volunteers demonstrating the translation of microbial composition from preclinical models to humans as well as harnessing the plasticity in the microbiota across human diversity. Furthermore, in association with dietary intake, the microbial module demonstrated significant correlation with the dietary habits of healthy adults living in diverse geographical locations and children being formula vs breast-fed. Our study provides a conceptual platform to translate microbial ecology from preclinical models to human populations.

## Materials and Methods

Public dataset processing: The datasets presented in this study were downloaded from the NCBI SRA database and MG-RAST, the datasets have been summarized in Table S4, including the accession numbers and associated publications. In case of sequences from the NCBI SRA, the datasets were downloaded as sra files and using the protocol described at the SRA site, the sequences were converted to fastq files, using the sra toolkit, deploying the fastq-dump command. In case of MG-RAST, the processed fasta files containing 16S sequences pre-screened for 97% identity to ribosomal genes were retrieved.

The resulting fasta files derived from the two locations were then analyzed identically using the software mother, the primary data cleaning steps were used as described in the 454 SOP. The computational environment was a server running 64 bit Red Hat Enterprise Linux release 5 with a 64 bit mothur ver 1.24 (CentOS precompile version) from the mothur website (http://www.mothur.org/wiki/454_SOP. Accessed 2013 July 1) [6]–[7]. Specifically the trim.seqs commands with oligos files generated from barcodes present on the NCBI page of the respective sequences. This removed any sequences with ambiguous bases, homopolymers longer than 8 and allowed for barcode difference of one base and a primer difference of two bases. The sequences post quality check and grouping by barcode were assigned taxonomies using the latest RDP template (release 7) from mothur website (http://www.mothur.org/wiki/RDP_reference_files. Accessed 2013 July 1). The command classify.seqs used was with 1000 iterations per sequence.

**Figure 1:**
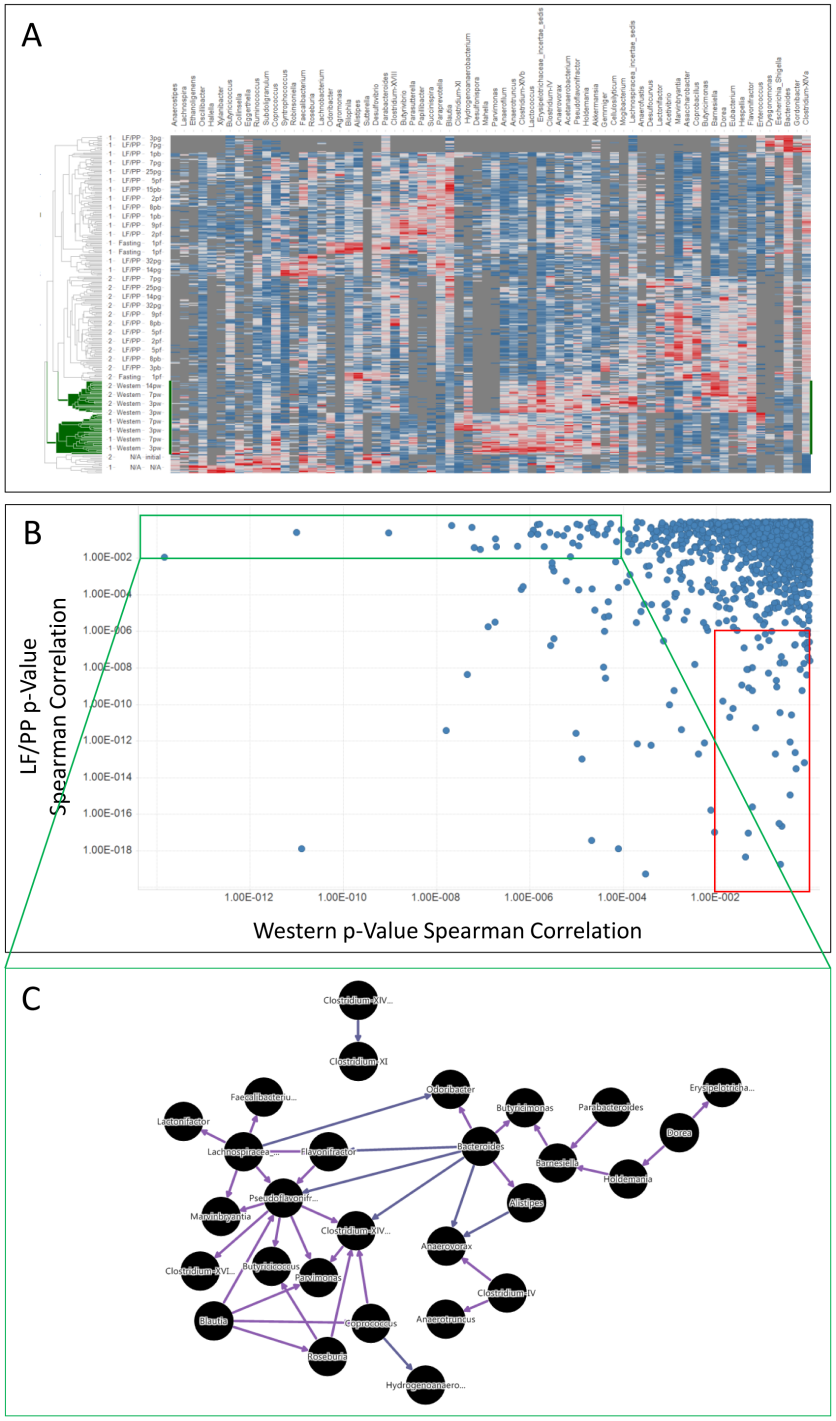
Detection of Diet-associated Ecological Communities. **A**. Hierarchical clustering of fecal 16S rRNA sequences in samples derived from humanized gnotobiotic mice. On the y-axis the human donor (1 or 2), diet (LF/PP; low-fat plant polysaccharide-rich mouse chow, Western; Harlan-Teklad TD96132, Fasting; no food, N/A; human sample prior to inoculation) and time point (indicated by day following dietary change and condition; N/A; human sample cultured prior to inoculation, initial; human fecal sample prior to inoculation, pw; post-Western diet change, pf; post fasting, pg; post-gavage, pb; post-return to mouse chow). On the x-axis the composite bacterial genera are indicated. Heat map; red (high abundance), blue (low abundance), grey (none detected). Hierarchical clustering for both rows and columns was performed using UPGMA clustering method with Euclidean distance measure, ordering weight by average value, normalization on a scale between 0 and 1 with empty value replacement by constant value given as 0. **B**. Identification of diet-related genera/genera interactive pairs. Pairwise genera p-values were calculated by co-occurrence analysis using Spearman correlation. Each point on the graph represents the relationship between two bacterial genera plotted across the p-value observed in the Western diet and the LF/PP (low fat, plant polysaccharide rich)-associated diets. The resulting Western diet-specific (green box) and LF/PP diet-specific (red box) pairs were selected for further analysis. **C**. Network map of Western diet-specific bacterial interactions. The Western diet-specific pairwise associations identified in B (green box) were assembled to visualize the diet-associated ecologic module. Purple edges indicate positive Spearman correlations, blue indicate negative correlations.

Data Analysis. Excel files (Tables S5–S8) containing the summarized counts of classified sequences derived above were manually annotated with information from publication or databases, this was done as the deposited data did not have enough metadata on them. This required to cross matching and subsequently merging the data from the associated publication like diet types, breakdown of dietary components etc and deposited metadata with the sequences. The resulting summary files are attached in the Supplementary Materials (Tables S5–S8) for reproducibility. The genus frequency/sample was calculated to normalize for differences in the number of sequences in each sample, which essentially meant deriving the percentage distribution of each genus representation within a sample. The resulting files were imported into TIBCO Spotfire 3.3.2.5 for all data normalizations, calculations, and statistical operations including visualization. Genus to Genus Spearman correlations as seen in figure 1B and Table S1 were calculated with 0 counts removed and cutoffs applied using the Df's calculated within the correlations as indicated.

## Results

### Identifying A Western Diet Ecological Module In Mice

We considered the concept that the microbial composition of a given ecosystem is the product of multiple dynamic and interactive bacterial populations (or modules) [8]–[10]. We sought to identify ‘modules’ of bacteria associated with dietary intake by examining statistical association of individual genera in a study examining association of the Western diet with the microbiota in humanized gnotobiotic mice [5]. This study investigated ‘humanized’ mice by using fecal transplants from human donors in germ free mice, which were later fed on Western and normal diet, and also included a diet crossover as part of the experimental design. The study aimed at reproducing microbial flora in a surrogate host (mice) and look at microbial flora perturbation following diet changes. The raw 16S sequences were classified and 70 genera of were included in subsequent analysis having been present in ≥25% of the 300 fecal samples. We did not consider culturing prior to inoculation as a factor as the original study did not detect a significant impact on the overall composition of the microbiota. We processed the annotated 16S rRNA sequences derived from two human donors grafted into germ-free mice including 70 genera for further analysis [11].

Examining the composition of individual samples at the Genera level (Figure 1A) samples derived from mice receiving the Western diet (in green) could be distinguished from mice receiving normal chow consistent with the original observations of the authors [5]. In addition, donor-dependent clustering dominated the grouping of the samples supporting the concept of an innate microbial ‘enterotype’ [3], [4]. As anticipated with a Western dietary switch, a trend towards time-dependent segregation of the samples was also observed. The bacterial composition at Western diet day 7 and 14 generally associated independently from the day 1 and 3 samples. The samples collected following reversion back to mouse chow were generally intermingled with those collected prior to the switch to Western diet, reflecting a plastic but predictable dynamic of the microbial composition in response to their environment.

Having confirmed the presence of diet-associated dynamic changes in the microbiota, we examined the pair-wise Spearman correlations of all 70 genera to identify co-segregating bacteria, hypothesizing that they would form the building blocks of ecologic modules associated with diet. Generic pairs significantly associated independent of diet, exclusively with Western diet and excluded from Western diet were all identified (Figure 1B, Table S1). For example, *Marvinbryantia* demonstrated a significant correlation with *Lachnospiracae insertae sedis* in the Western diet vs mouse chow (p = 1.45e^−14^ vs p = 1.14 e^−2^) and similarly *Alistipes* abundance correlated with *Bacteroides* by Spearman correlation (p = 9.79e^−12^ vs 2.69e^−1^). Furthermore, *Clostridium XVIII* and *Coprobacillus* had a Spearman correlation of 1.78e^−19^ in the mouse chow diet (LF/PP) vs 2.36e^−1^ in Western diet samples. These data demonstrate that diet influences the coordinated association of bacteria with each other. This follows conventional wisdom regarding bacterial interaction that interactions between individual bacteria can be synergistic or antagonistic in response to competition for food or the interdependence of groups of microbes to metabolize and/or utilize particular nutrients.

We next investigated the integration of the Western diet-specific pairs into potential ecological modules via building a network map (Figure 1C). The Western diet-associated pairs demonstrated a marked degree of integration. One module of 9 positively correlating genera was observed centered on *Bacteroides* – the focus of one of 3 proposed ‘enterotypes’ [3], [4]. This potential module negatively-associated with a second module of 13 genera surrounding *Pseudoflavonifractor*. Two additional modules (3 genera negatively-associated with the *Bacteroides* module and a fourth, single interaction between *Clostridium XIVa* and *Clostridium XI*) were also observed, however, we focused our attention on those surrounding *Bacteroides* and *Pseudoflavonifractor*.

**Figure 2:**
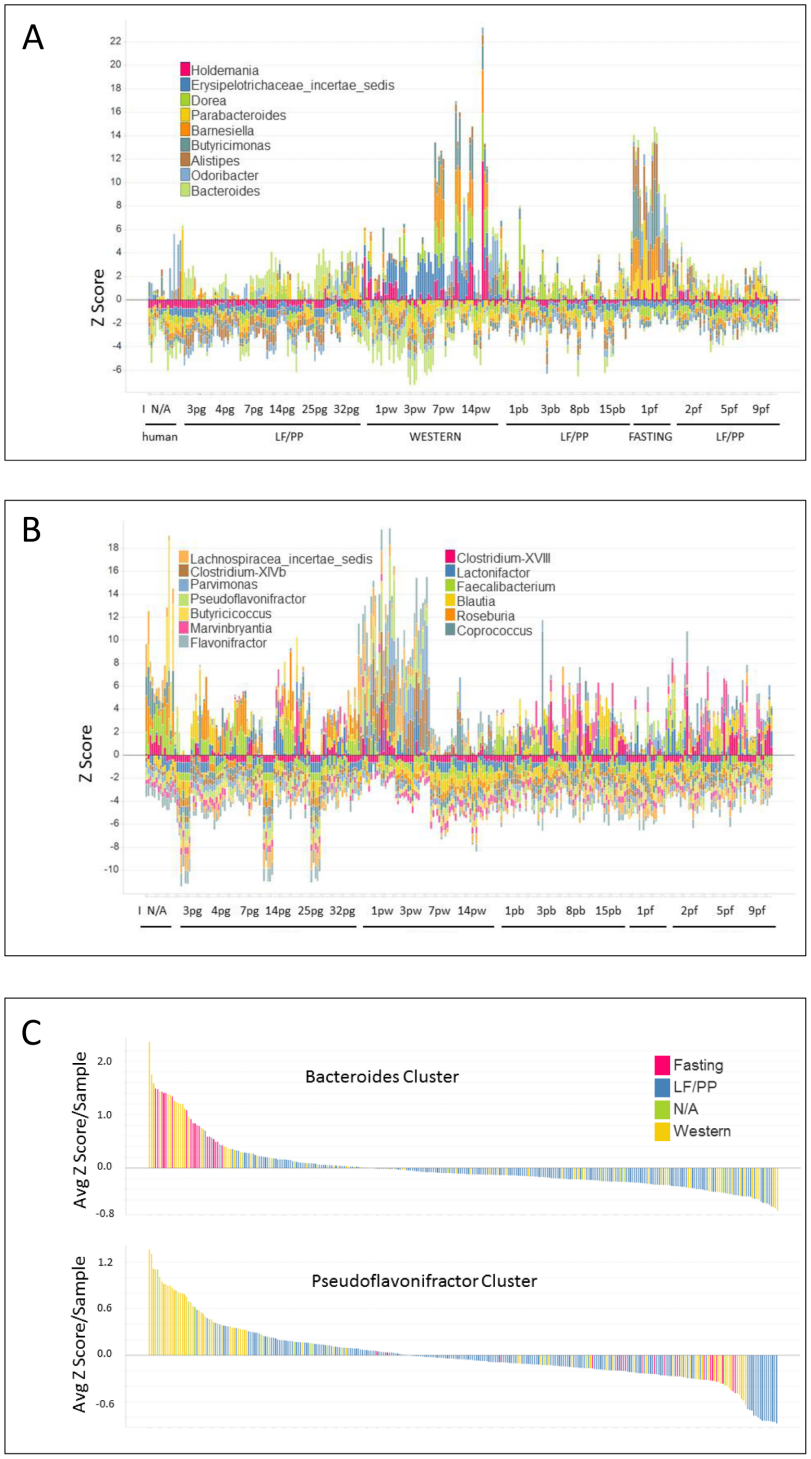
Temporal and diet-regulated dynamics of ecological bacterial modules. **A**. Bacteroides module. Each stacked bar represents the Z score normalized abundance of the component genera indicated in the legend. The diet and time points are indicated on the x-axis and can generally be followed in a temporal manner from left to right. Diets shown are human (undefined starting material), LF/PP (low fat/plant polysaccharide rich chow), WESTERN (Harlan-Teklad TD96132), FASTING (no food). I; initial fecal donor sample, N/A; initial sample prior to inoculation, pg; day following gavage into mouse, pw; day following shift to Western diet, pb: day following return to LF/PP diet, 1pf: day 1 fasting, pf: day after fasting. **B**. Temporal and diet-regulated dynamics of the *Pseudoflavonifractor* module. Each stacked bar represents the Z score normalized abundance of the component genera indicated in the legend. X-axis as for A. **C**. The abundance of the *Bacteroides* and *Pseuodoflavonifractor* modules associate with Western-diet. The average Z score for each fecal sample was calculated for both the *Bacteroides* and *Pseudoflavonifractor*-module component genera and arranged in order of abundance from left to right. Each bar is coloured by the sample diet.

The next logical step was to investigate whether the identified potential modules reflected dietary intake. We normalized abundance levels of the composite genera of the two potential modules using a Z score calculation to consider each genus with equal weighting and plotted these across the individual samples (Figure 2A, 2B). The abundance of the individual genera comprising the *Bacteroides* module was elevated in the latter Western diet samples (7 and 14 day; 7pw, 14pw) and later the ‘fasting’ samples when mouse chow was withdrawn for 24 hours (Figure 2A). In both cases genera abundance returned to prior levels when the LF/PP diet was re-initiated suggesting the overall abundance of the module was inhibited by the LF/PP diet. The component genera of the *Pseudoflavonifractor* module demonstrated the opposite temporal pattern being elevated at early time points (day 1 and 3) following the shift to Western diet then decreasing thereafter (Figure 2B). The negative correlation between the two modules is derived from the differential abundance of the modules over time following the switch to Western diet. While this observation could indicate that the *Bacteroides* module represents a ‘mature’ Western dietary module, both modules demonstrated partitioning with the Western diet. Therefore, we continued to investigate both mature *Bacteroides module* and the *Pseudoflavonifractor module* in our analysis.

As an overall reflection of abundance of the modules, we next calculated the average Z score/sample and assembled the samples in order for both the *Bacteroides* and *Pseudoflavonifractor* modules (Figure 2C). Annotated Western diet samples (yellow) were enriched towards the left of the respective module graphs reflecting an association of the abundance of both modules with the Western diet.

We also generated a network from the pairwise genera associations that were specific to the samples derived from the mouse chow diet (Figure 1B; red box, Figure S1). A central module of 12 genera around a *Barnesiella* hub was observed surrounded by 4 negatively correlating minor modules. *Blautia, Syntrophococcus* and *Clostridium XIVb* formed 7, 4 and 4 negative connections, respectively with peripheral members of the *Barnesiella* module. The *Barnesiella* module itself showed no association with diet but did distinguish samples derived from donor 2 (Figure S2). The phenotype of this module, therefore, support the concept of an innate individual enterotype more than an association with diet (Figure 1B, Table S1, Figure S2).

### The Bacteroides Module Stratifies Human Samples According To Western Diet

**Figure 3:**
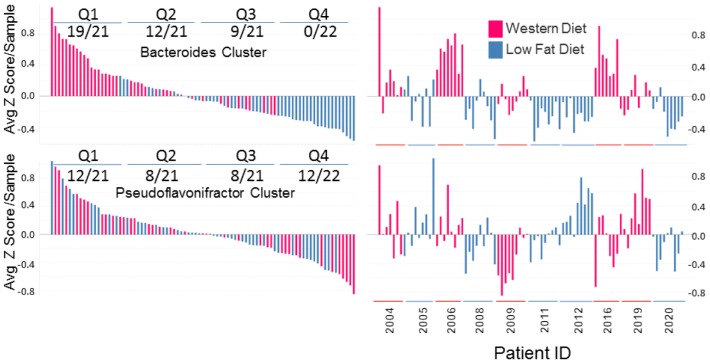
The *Bacteroides* module is associated with Western diet in human patients. On the left is plotted the average Z score for each sample and placed in order of decreasing abundance from left to right. The quartile distribution of high fat diet samples/total is shown for each module. On the right the same average Z scores are plotted with annotation from each patient. The temporal order for each patient is from left to right. Only samples on a controlled Western or low-fat diet (as indicated) were included in the analysis – starting samples prior to commencing the controlled diet were excluded.

Since the original study was derived from a mouse model inoculated with a human microbiota, we hypothesized that if the modules were physiologically relevant to diet, human subjects receiving a similar diet should be distinguished by their relative average Z score for the module (Figure 3). To do this, we turned our attention to another published study examining the dynamic properties of a controlled dietary change on healthy volunteers [3]. In this particular study healthy human subjects were fed in clinical settings using a high fat/low fiber or a low fat/high fiber diet, which represented a Western and traditional diet, respectively. This was followed up by examination of the feces for microbial perturbation as a result of the diet. The average Z score for the *Bacteroides* and *Pseudoflavonifractor* modules was calculated for the annotated human samples where the *Bacteroides* module demonstrated an enriched distribution in the first quartile correlating with 19/21 samples and a complete absence in the last quartile. In addition, samples from all 5 patients on the high fat/low fiber diet were identified in the top quartile and samples from all 5 patients on the low fat/high fiber diet were identified in the bottom quartile. The *Pseudoflavonifractor* module that associated with donor 2 rather than dietary intake in mice, likewise did not stratify the patient population according to fat intake (Figure 3). These data demonstrate that microbial ecology of preclinical models can predict human populations independently of a variance ‘enterotype’ approach.

**Figure 4:**
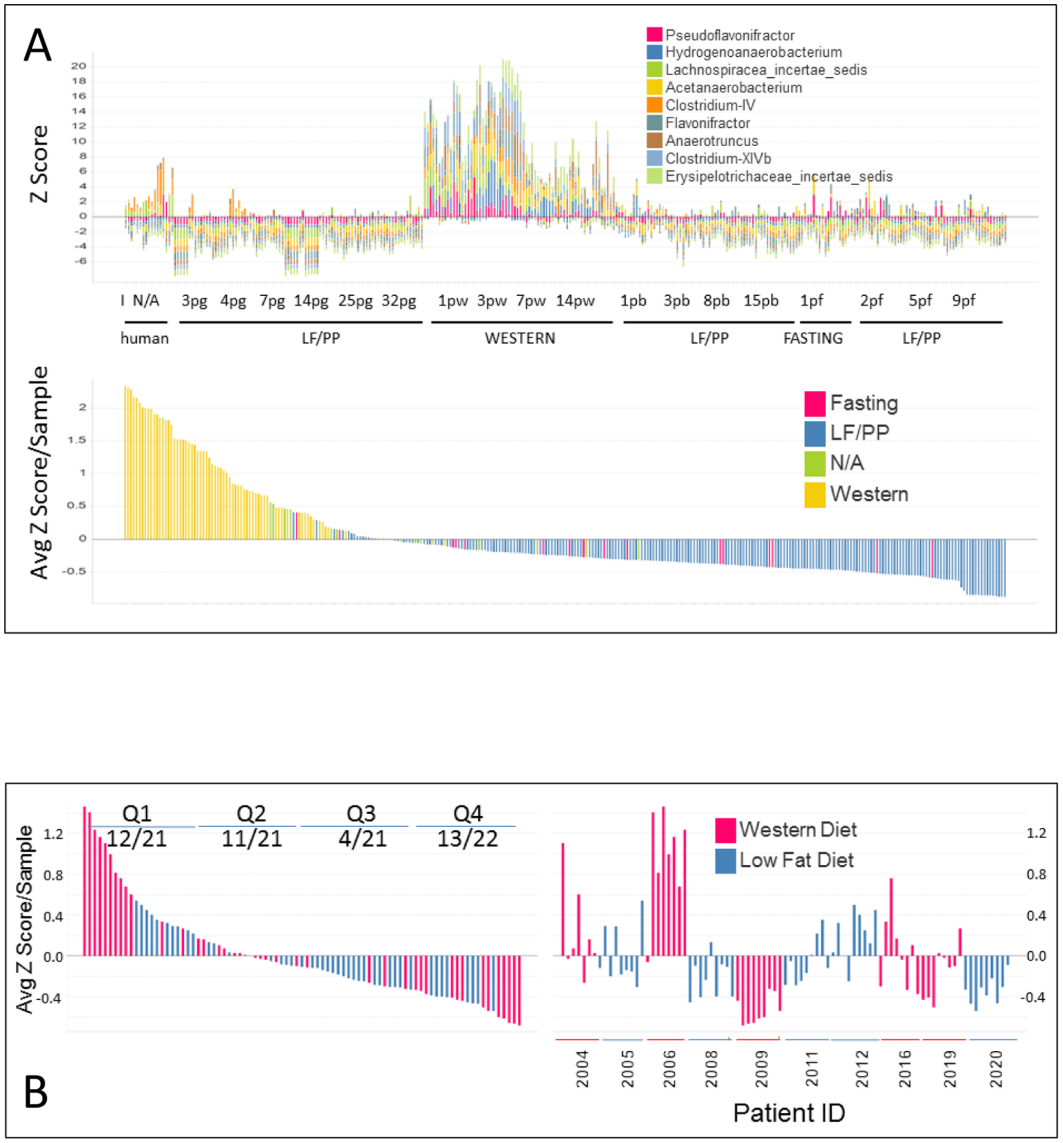
Bacterial abundance in humanized mice is a poor predictor of dietary intake in humans. **A**. Dynamic association of control ‘abundance module’ profile with dietary intake in the mouse. The top panel displays the Z score abundance of each of the genera indicated across time and dietary intake in the humanized mouse. The bottom panel shows the average Z scores for each fecal sample placed in order and coloured according to dietary intake of the mouse. **B**. The control ‘abundance module’ derived from humanized mice does not associate with diet in a controlled human study. Annotation is the same as Figure 3A.

We tested whether the association of module abundance with the Western-diet samples simply reflected the individual genus abundance in each sample. In the *Bacteroides* module, the abundance of 6 out of 9 of the members were significantly associated with Western diet in humans – of these only *Holdemania* was significantly lower in the high fat diet samples. In the *Pseudoflavonifractor* module, 6 out of 13 genera were significantly associated with diet. Three of these (*Blautia, Flavonifractor* and *Butyricoccus*) were significantly higher in the patients on a low fat diet. One possibility for the association between the gnotobiotic mouse and human study was simply a result of identifying generally high abundance genera in the humanized mice. To examine this, we identified the top 9 genera most significantly associated with Western diet in the humanized mouse study (Table S2). Unsurprisingly, the gnotobiotic mouse samples were partitioned according to diet by the average Z score of this ‘Western Abundance module’ (Figure 4A). However, when the average Z score of this module of bacteria was applied to the human dietary study it did not associated with diet (Figure 4B). Although the top 10 most abundant samples were all high fat diet-associated, 7 out of 10 were derived from 1 patient and the top quartile contained only 12/21 high fat-diet samples. In addition, the bottom quartile was populated by 13/22 high fat diet samples mostly from two patients, clearly indicating that this ‘abundance’ module could not distinguish diet-related changes from the variability inherent between individuals. Only 3 of the ‘abundance’ genera were also significantly different in the human study (*Hydrogenoanaerobacterium, Anaerotruncus, Clostridium IV*) while *Flavonifractor* was significantly lower in the human high fat vs low fat diet samples. Therefore, abundance of bacterial genera in the absence of ecological context is a poor predictor of the diet-related dynamics of microbial ecology.

### The Bacteroides Module Correlates With Dietary Intake In The General Population

The data shown above demonstrate that the *Bacteroides* module can distinguish samples derived from a Western diet under control conditions in both mouse and human. Thus it stands to reason that if the *Bacteroides* module was a biomarker of dietary intake the abundance of the module should correlate with dietary intake in the general population. To test this, we examined another dataset in a human study examining the composition of the microbiota with two dietary questionnaires; one recording patients' recent diet (Recall) and another querying habitual long term diet (FFQ). The study investigated the effect of long term diet and its effect of microbial composition using questionnaires of the habitual long term diet. It was suggested that enterotypes are a result of long term dietary habits and when it is perturbed for a short period using a diet intervention the core enterotype remains the same. Using the *Pseudoflavonifractor* and ‘Western Abundance’ modules as controls, the association of the *Bacteroides* module with dietary intake was determined. A total of 24 individual dietary factors from the two questionnaires correlated with the *Bacteroides* module average Z score with a p value <0.01 (Table 1). This compared with no significant correlations found in either the *Pseudoflavonifractor* or the ‘Western Abundance’ modules. From the ‘Recall’ questionnaire, positive correlation of the *Bacteroides* module was observed with 6 individual saturated fatty acids (SFA), total saturated fatty acids and percent calories from saturated fatty acids. Negative correlation was seen with the ‘Polyunsaturated to Saturated fat ratio’, as well as forms of the vegetable-enriched fat-soluble Vitamin E consistent with the emphasis of the Western diet on animal vs vegetable fats [12]. Again, related to the Western shift away from fruit and vegetable intake, negative correlations with the complex carbohydrate pectin, Vitamin C and dietary fiber were also observed consistent with the conclusion that the *Bacteroides*-associated bacterial module is a Western diet-associated dietary ecological module. These data demonstrate that an ‘ecological’ view of the microbiota can overcome the challenges associated with microbial plasticity and variation in individual populations. This suggests that a similar approach has potential to translate microbial ecology into the clinical setting.

**Table 1:** Diet Intake Correlation with Ecological Module: RECALL and FFQ Questionnaires [3].

	*BACTEROIDES*		*HIGH ABUNDANCE*		*PSEUDOFLAV-ONIFRACTOR*		
*Dietary Component*	*p-Value*	*r*	*p-Value*	*r*	*p-Value*	*r*	*Quest.*
Vitamin.E.IU.	3.93E-04	−0.3511	8.29E-02	−0.1761	8.44E-01	0.0201	RECALL
Vitamin.E.mg.	4.57E-04	−0.3474	5.91E-02	−0.1913	9.78E-01	0.0029	RECALL
Total.Alpha.Tocopherol.Equivalents	4.67E-04	−0.3469	1.12E-01	−0.1617	7.48E-01	0.0329	RECALL
Phytic.Acid	6.38E-04	−0.3390	1.27E-01	−0.1550	8.00E-01	−0.0260	RECALL
Polyunsaturated.to.Saturated.Fat.Ratio	6.59E-04	−0.3382	7.24E-01	−0.0362	1.82E-02	0.2382	RECALL
Pectins	1.05E-03	−0.3260	2.77E-01	−0.1110	4.62E-01	0.0751	RECALL
Vitamin.C	1.14E-03	−0.3239	1.17E-02	−0.2536	7.12E-01	0.0378	RECALL
Total.Saturated.Fatty.Acids.SFA	1.18E-03	0.3231	3.42E-01	0.0969	4.03E-01	−0.0854	RECALL
Percent.Calories.from.SFA	1.26E-03	0.3212	6.49E-01	0.0466	1.15E-01	−0.1603	RECALL
SFA.myristic.acid	1.73E-03	0.3126	1.23E-01	0.1569	3.91E-01	−0.0877	RECALL
SFA.butyric.acid	2.42E-03	0.3030	2.67E-01	0.1131	6.68E-01	−0.0439	RECALL
glu (glucose)	2.60E-03	−0.3010	3.87E-01	0.0883	4.96E-01	−0.0696	FFQ
Insoluble.Dietary.Fiber	2.79E-03	−0.2989	2.00E-01	−0.1307	9.53E-01	0.0060	RECALL
SFA.caproic.acid	3.15E-03	0.2954	3.00E-01	0.1057	6.38E-01	−0.0482	RECALL
SFA.stearic.acid	3.99E-03	0.2883	6.97E-01	0.0398	2.51E-01	−0.1171	RECALL
Synthetic.Alpha.Tocopherol	3.99E-03	−0.2883	4.15E-02	−0.2063	5.78E-01	−0.0569	RECALL
Total.Dietary.Fiber	4.34E-03	−0.2858	1.59E-01	−0.1432	9.35E-01	0.0084	RECALL
SFA.palmitic.acid	5.03E-03	0.2812	7.01E-01	0.0392	6.31E-01	−0.0492	RECALL
f140 (Myristic fatty acid)	6.15E-03	0.2749	6.14E-01	0.0516	2.46E-01	−0.1183	FFQ
Natural.Alpha.Tocopherol	7.65E-03	−0.2679	9.91E-02	−0.1675	8.55E-01	−0.0187	RECALL
fruct (fructose)	7.87E-03	−0.2670	2.97E-01	0.1064	3.60E-01	−0.0935	FFQ
SFA.capric.acid	8.06E-03	0.2662	2.11E-01	0.1275	3.63E-01	−0.0929	RECALL
t161 (Palmitoleic fatty acid)	8.11E-03	0.2660	7.83E-01	0.0282	4.80E-01	−0.0722	FFQ
Total.Conjugated.Linoleic.Acid.CLA.18.2	8.16E-03	0.2658	9.58E-01	−0.0054	1.83E-01	−0.1357	RECALL

Significant (p<0.01) Spearman correlations of ‘*Bacteroides*’ module abundance (Average Z Score) with dietary intake as reported in COMBO. No significant correlations were found for either ‘High Abundance’ or ‘*Pseudoflavonifractor*’ modules. See Table S3 for complete dataset.

### The Bacteroides Module Correlates With Dietary Intake Independent Of Geographical Location

Having established the connection of the *Bacteroides* module with the Western diet in mice, a controlled Western diet study in humans and with recent dietary intake in a Western population, we sought to establish whether it also reflected the dietary habits in diverse geographical locations. To answer this we applied the concept to a recently published study that investigated the fecal microbiota composition across geography by comparing three populations; a Western population from the USA, a population of Guahibo Amerindians residing in Venezuela, and a third population from rural communities in Malawi [13]. The study was comprised of 531 individuals including healthy adults and children, geographical location and their dietary habits. The findings reported similar functional and microbial components in early life, maturing over the first three years of early life depending on local dietary habits. This study demonstrated the common theme of the dietary influence on a stable microbial ‘enterotype’ that distinguished USA volunteers from those of the developing and underdeveloped world.

**Figure 5:**
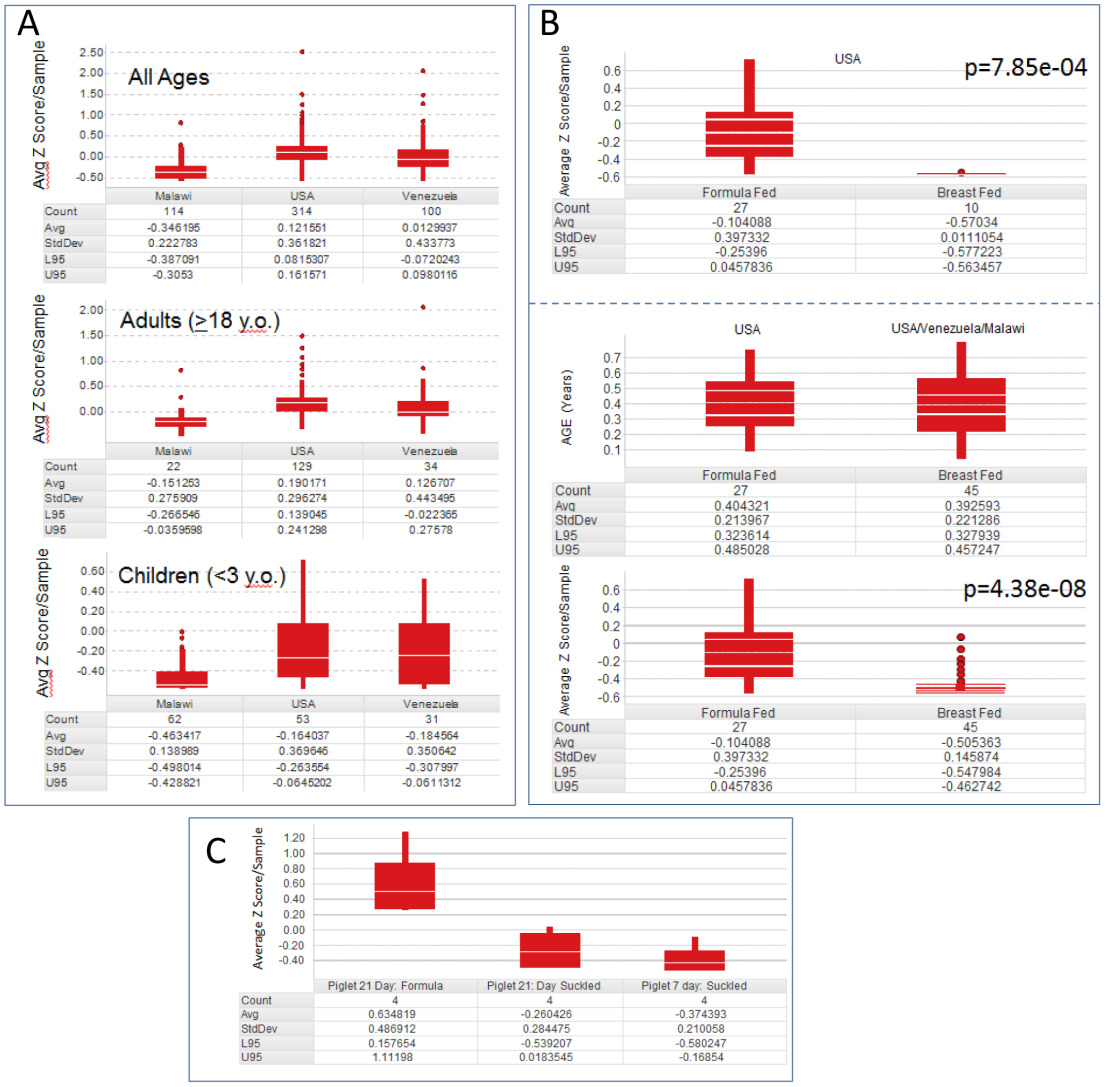
The Bacteroides module distinguishes geographical dietary intake and formula feeding in children and piglets. **A**. The *Bacteroides* module is endemic to all ages of the developed and developing world. The average Z score per fecal sample was calculated and combined to compare the three populations. The number of samples included with the given criteria (Count), average of the calculated Z scores/sample (Avg), the standard deviation (StdDev), and the lower 95% and upper 95% confidence limits (L95 and U95, respectively) are displayed in the Tables. The top panel includes all available samples in the study, regardless of age annotation. The middle panel includes only adults with an annotated age 18 years old or more. The bottom panel includes children with an annotated age 3 years old or less. In all cases, the Malawi samples have significantly lower abundance of the *Bacteroides* module than those from the USA and Venezuela. **B**. The Western diet-associated *Bacteroides* module also associates with formula feeding in children. Average Z scores per sample were calculated as in A. The p-values for *Bacteroides* module Z score association with diet by ANOVA are indicated. Top panel: all samples from the USA population that were annotated for formula or breast feeding were included in the analysis. Middle panel: Distribution of ages of annotated breast vs formula fed samples. Age matched samples all ≤0.8 years old (as indicated by top box plot) were analyzed. Only USA samples were annotated as formula fed as indicated. Bottom panel: The association with formula feeding remained significant in age matched samples across populations. **C**. The *Bacteroides* module associates with formula feeding in piglets as well as humans. Piglets suckled for 7 days (right) were switched to formula for 14 days (left), or left to suckle an additional 14 days (middle). The average Z score for the *Bacteroides* module from each sample was plotted.

The abundance of the *Bacteroides* module was lowest in the Malawian population correlating with a low fat/high fiber diet. However, no significant difference in the abundance of the *Bacteroides* module was found between the Venezuelan and USA populations (Figure 5A). The divergent segregation of the Venezuelan and Malawian samples based on the *Bacteroides* module was supported by the responses to the diet questionnaires contained in the supplementary data of the report [13]. The Venezuelan, but not the Malawian, family food intake included frequent consumption of ‘Soda,’ ‘Milk,’ ‘Butter,’ canned meat and fish, ‘Cheese,’ and other foods that are perhaps more commonly associated with a Western diet [13]. For example, one Malawi family (of 28 families in total) reported ‘thobwa’ (a local millet-based drink) and ‘soda’ use twice daily Indeed, blog references were found of ‘thobwa’ being referred to locally as ‘soda’ (http://canteriointernational.org/blog4/2010/06/15/togba-malawi-local-soft-drink. Accessed 2013 July 1). This compared with 19/56 Venezuelan individuals reporting ‘Soda’ consumption at least once daily including 4/14 children less than 3 years old. Therefore, the epidemiology of the *Bacteroides* module correlated with Western dietary intake independently of geographical location.

### The Bacteroides Module Correlates With Dietary Intake (Formula Vs Breast Feeding) In Children

Using the annotation provided with the geographical study, we investigated the association of the *Bacteroides* module with another dietary factor, namely the Western influence of formula vs breast-feeding in children. The development over time of the *Bacteroides* module was firstly investigated using the annotation provided for the USA vs Venezuela vs Malawi study [13]. Consistent with the conclusions of the original study, there was a significant association of the module with age independent of the country of origin likely resulting from the maturation of the gut flora in young children less than 3 years old (Figure S3). The use of formula or breast feeding was also annotated in the original study. Therefore, we examined the association of the *Bacteroides* module with breast feeding in the USA population during this critical developmental period [13]. Children who were formula fed vs breast fed had significantly elevated levels of the *Bacteroides* module (Figure 5B). The association remained significant when age-matched samples (all samples ≤0.8 years old) were included in the analysis from the breast-fed Malawi and Venezuelan children.

In the published annotation, all of the Malawian and Venezuelan children less than 3 years old were breastfed. Malawi has relatively high reported breastfeeding rates contrasting with Venezuela with one of the lowest in the world [14]. Therefore, we critically questioned our observation associating the *Bacteroides* module with formula feeding. In order to ensure that this observation was not an artifact of age, geography or errors in annotation, we therefore decided to extend our analysis to other studies investigating the impact of breast vs bottle feeding on the composition of the microbiota.

Several candidate human studies were investigated, however, the abundance of the component genera making up the *Bacteroides* module were generally rare or absent (due in part to the young age of the participants and in part a lack of depth in the coverage of the sequencing or variable formats such as DGGE or qPCR) making a comparable assessment of our observations in available human datasets impossible. We decided therefore to turn our attention to a preclinical study examining microbiota changes in response to a formula diet in piglets [10], [15]. Here piglets were fed formula milk without access to breast milk and their gut microflora was contrasted with the control suckled animals. The data was processed identically as for the human studies determining an average Z score/sample. The highest abundance samples were all derived from the piglets on a formula diet and this was reflected by the significant difference in Z score between the formula fed vs milk fed piglet groups by ANOVA (Figure 5C). Therefore, in a controlled experimental preclinical setting as well as the human populations, the *Bacteroides* module associates with formula feeding. These data support the association of the module with dietary intake across organisms, age and geography.

## Discussion

The conclusions from this study demonstrate several important principles. Firstly, preclinical models can be used to predict human biology responding to an often-leveled criticism of microbiota-based studies [15]. While arguments can be made regarding the best preclinical model to use to translate findings into human populations, the technical expertise, infrastructure, and resources surrounding genetically modified mice means they remain the preferred choice for basic and translational research. The conclusive data presented here provide a paradigm to identify biologically-relevant ecological modules in humans. It is reasonable to assume that the approach taken, in this case to investigate Western diet, could equally be applied to identifying bacterial ecology associated with aspects of host genetics, pharmaceutical treatment, drug metabolism and other applications, simplifying the complexities inherent in the analyses of ‘top-down’ approaches. However, while specific associations with dietary intake have been demonstrated here in the general population, it remains to be tested what metabolic function(s) the *Bacteroides* module might have in this context.

There has been extensive interest in the impact of globalization and its associated dietary influences with the increasing economic and human burden of chronic diseases (such as obesity and diabetes) in the developing world. Death rates from chronic diseases surpass those from infectious diseases in every continent of the world except for Africa [1]. We observed a similar pattern in the association of the *Bacteroides* module with dietary intake in geographical populations. Although our preliminary analysis did not detect any correlation with BMI or other annotated factors independent of diet, it will be interesting to determine whether the *Bacteroides* module also correlates with chronic disease or outcome. Such a link would provide a path from the epidemiology of diet and chronic diseases, microbial ecology in preclinical models progressing into human populations, and provide a clear strategy for identifying therapeutic interventions. In addition, a similar approach may also provide insight into other interventions outside of diet, for example, recovery of the gut flora following antibiotic or NSAID usage. It will also be interesting to identify the critical members of such self-sufficient networks and the degree of perturbation they can resist before the network collapses.

The impact of breast vs bottle feeding on human health is an important and emotive issue [16], [17]. While there is general consensus that exclusive breastfeeding helps to prevent infectious diseases, obtaining a clear association with the risk of chronic diseases has been confounded by a number of factors although many studies have highlighted an association of breastfeeding with a decreased odds-ratio of childhood obesity as well as risk factors for cardiovascular diseases in adulthood [18], [19]. We demonstrate that the *Bacteroides* module associated with a Western diet in adults is also associated with formula (vs breast) feeding in young children. In adults, there was a significant positive association of the module abundance with saturated fatty acids including linoleic and capric acid intake as well as the ratio of polyunsaturated fatty acids to saturated fatty acids (Table 1). These observations parallel the fatty acid composition over the course of lactation and provide a link between our observations with the *Bacteroides* module, the development of the microbiota and the impact of formula feeding [20], [21]. The results of our study come in context of the continuing debate regarding dietary supplementation to infants, questions regarding the link of Western dietary habits to the rising prevalence of chronic diseases and the recently considered role of the microbiota underlying their association.

The concepts that we have used in this study are recognized standards in microbial ecology in other ecosystems such as fresh water lakes, oceans and soil. In these ecosystems, microbes are viewed not as singular life forms working in isolation and complete independence but as interacting and interdependent groups. We have applied these established principles to identify a dietary microbial module that can be translated from preclinical models to humans. Similar microbial correlations with dietary intake may also underlie the association of diet and the chronic disease epidemic and further studies will determine whether this is the case.

## Supporting Information

Figure S1 **Network map of LF/PP diet-specific bacterial interactions.** The LF/PP diet-specific pairwise associations identified in Fig.1B (red box) were assembled to visualize the diet-associated ecologic module. Purple edges indicate positive Spearman correlations, blue indicate negative correlations.
(TIF)

Figure S2 **The abundance of the** ***Barnesiella*** **cluster associates with human donor rather than diet.** An average Z score of the *Barnesiella* cluster component genera was calculated for all fecal samples and arranged in order of abundance from left to right. Top panel: diet related annotation by colour. Bottom panel: donor related annotation as indicated.
(TIF)

Figure S3 **The Bacteroides cluster abundance is developmentally regulated.** The Spearman correlation of average Z score/sample with age is shown for the three populations studied. Rank ordering of age was performed with all three populations pooled for comparison. The equation of the linear correlation is shown with the r^2^ correlation and p-value for each population.
(TIF)

Table S1 ‘Genus to Genus’ Spearman correlations: Western diet vs LFPP. Summary table of calculated statistics of correlated pairwise genera abundance in either Western or LFPP diet-annotated samples from humanized gnotobiotic mice. For the analysis summarized in Fig.1B only pairwise associations with at least 50 Df in the LFPP diet and 16 Df in the Western Diet were considered.
(TXT)

Table S2 Genus abundance ANOVA Statistics: Western diet vs LFPP. Summary table of ANOVA calculated p-values identifying statistically different abundance of genera vs diet. Genera contained within Bacteroides, Pseudoflavonifractor and ‘Western Abundance’ clusters are indicated. N.S. Not Significant.
(TXT)

Table S3 Spearman correlations of *Bacteroides* cluster abundance (Avg Z Score) with dietary intake as reported in COMBO. All values recorded. Significant (p<0.01) associations shown in table 1. *Spearman correlation significant, however association is artifact of 0's in raw data.
(TXT)

Table S4 Original datasets, links and publications analyzed in this study.
(TXT)

Table S5 Summary file of Genus abundance with annotation from Gnotobiotic mouse study. Values shown are frequency per sample (count Genus/total counts).
(TXT)

Table S6 Summary file of Genus abundance with annotation from Human CAFE Diet Study. Both raw counts/sample (top) and Normalized values (frequency/sample: bottom) are shown.
(TXT)

Table S7 Summary file of Genus abundance with annotation from Human COMBO Dietary Intake Study. Both FFQ and Recall dietary annotation is included.
(TXT)

Table S8 Summary file of Genus abundance with annotation from Human Age and Geography study. Both raw counts/sample (top) and normalized values (frequency/sample: bottom) are shown.
(TXT)

Table S9 Summary file of Genus abundance with annotation from formula-fed piglets. Both raw counts/sample (top) and normalized values (frequency/sample: bottom) are shown.
(TXT)

## References

[1] World Health Organization (2003) Diet, Nutrition and the Prevention of Chronic Diseases: Report of a Joint WHO/FAO Expert Consultation. Geneva: World Health Organization.

[2] Cummings JH, Macfarlane GT (1997) Role of intestinal bacteria in nutrient metabolism. *JPEN* J Parenter. Enteral. Nutr. 21: 357–365. 9406136 10.1177/0148607197021006357

[3] Wu GD, Chen J, Hoffmann C, Bittinger K, Chen YY, et al (2011) Linking long-term dietary patterns with gut microbial enterotypes. Science 334: 105–108. 21885731 10.1126/science.1208344 PMC3368382

[4] Arumugam M, Raes J, Pelletier E, Le Paslier D, Yamada T, et al (2011) Enterotypes of the human gut microbiome. Nature 473: 174–180. 21508958 10.1038/nature09944 PMC3728647

[5] Goodman AL, Kallstrom G, Faith JJ, Reyes A, Moore A, et al (2011) Extensive personal human gut microbiota culture collections characterized and manipulated in gnotobiotic mice. Proc. Natl. Acad. Sci. USA 108: 6252–6257. 10.1073/pnas.1102938108 PMC3076821 21436049

[6] Schloss PD, Westcott SL, Tyabin T, Hall JR, Hartmann M, et al (2009) Introducing MOTHUR: open-source, platform-independent, community-supported software for describing and comparing microbial communities. Appl. Environ. Microbiol. 75: 7537–7541. 19801464 10.1128/AEM.01541-09 PMC2786419

[7] Schloss PD, Westcott SL (2011) Assessing and improving methods used in operational taxonomic unit-based approaches for 16S rRNA gene sequence analysis. Appl. Environ. Microbiol. 77: 3219–3226. 21421784 10.1128/AEM.02810-10 PMC3126452

[8] Little AEF, Robinson CJ, Peterson SB, Raffa KE, Handelsman J (2008) Rules of engagement: interspecies interactions that regulate microbial communities. Annu Rev Microbiol 62: 375–401. 18544040 10.1146/annurev.micro.030608.101423

[9] Strom SL (2008) Microbial ecology of ocean biogeochemistry: a community perspective. Science 320: 1043–1045. 18497289 10.1126/science.1153527

[10] Poroyko V, Morowitz M, Bell T, Ulanov A, Wang M, et al (2011) Diet creates metabolic niches in the “immature gut” that shape microbial communities. Nutr. Hosp. 26: 1283–1295. 22411374 10.1590/S0212-16112011000600015

[11] Smith P, Siddharth J, Pearson R, Holway N, Shaxted M, et al (2012) Host Genetics and Environmental Factors Regulate Ecological Succession of the Mouse Colon Tissue-Associated Microbiota. PLoS ONE 7: e30273. 22272321 10.1371/journal.pone.0030273 PMC3260280

[12] McCance R and Widdowson E (2002) Fats and Oils. In: McCance and Widdowson's the composition of foods. London: Royal Society of Chemistry. pp.131–143.

[13] Yatsunenko T, Rey FE, Manary MJ, Trehan I, Dominguez-Bello MG, et al (2012) Human gut microbiome viewed across age and geography. Nature 486: 222–227. 22699611 10.1038/nature11053 PMC3376388

[14] UNICEF (2007) *The state of the world's children 2008: child survival* (UNICEF Publication 978-92-806-4191-2, 2007; http://www.unicef.org/sowc08/docs/sowc08.pdf. Accessed 2013 July 1).

[15] Hvistendahl M (2012) Pigs as stand-ins for microbiome studies. Science 336: 1250. 22674332 10.1126/science.336.6086.1250

[16] Gordon JI, Dewey KG, Mills DA, Medzhitov RM (2012) The human gut microbiota and undernutrition. Sci Transl Med. 4: 137ps12. 10.1126/scitranslmed.3004347 22674549

[17] Fewtrell M, Wilson DC, Booth I, Lucas A (2011) When to wean? How good is the evidence for six months' exclusive breastfeeding. BMJ 342: c5955. 10.1136/bmj.c5955 21233152

[18] Koletzko B, von Kries R, Monasterolo RC (2009) Infant feeding and later obesity risk in *Early nutrition programming and health outcomes in later life: Obesity and beyond* (Springer, location 646 , 2009), B.Koletzko et al. eds., 15–29.

[19] Scientific Advisory Committee on Nutrition Subgroup on Maternal and Child Nutrition (SMCN) (2011) The influence of maternal, fetal and child nutrition on the development of chronic disease in later life, 2011 www.sacn.gov.uk. Accessed 2013 July 1.

[20] Brenna JT, Varamini B, Jensen RG, Diersen-Schade DA, Boettcher JA, et al (2007) Docosahexaenoic and arachidonic acid concentrations in human breast milk worldwide. Am J. Clin. Nutr. 85: 1457–1464. 17556680 10.1093/ajcn/85.6.1457

[21] Gibson RA, Kneebone GM (1981) Fatty acid composition of human colostrum and mature breast milk. Am.J.Clin.Nutr. 34: 252. 7211726 10.1093/ajcn/34.2.252

