# Supplementary figures and images for: A Western Diet Ecological Module Identified from the ‘Humanized’ Mouse Microbiota Predicts Diet in Adults and Formula Feeding in Children
Source: PLoS One. 2013 Dec 31;8(12):e83689. doi: 10.1371/journal.pone.0083689 (PMC3877084; doi:10.1371/journal.pone.0083689)

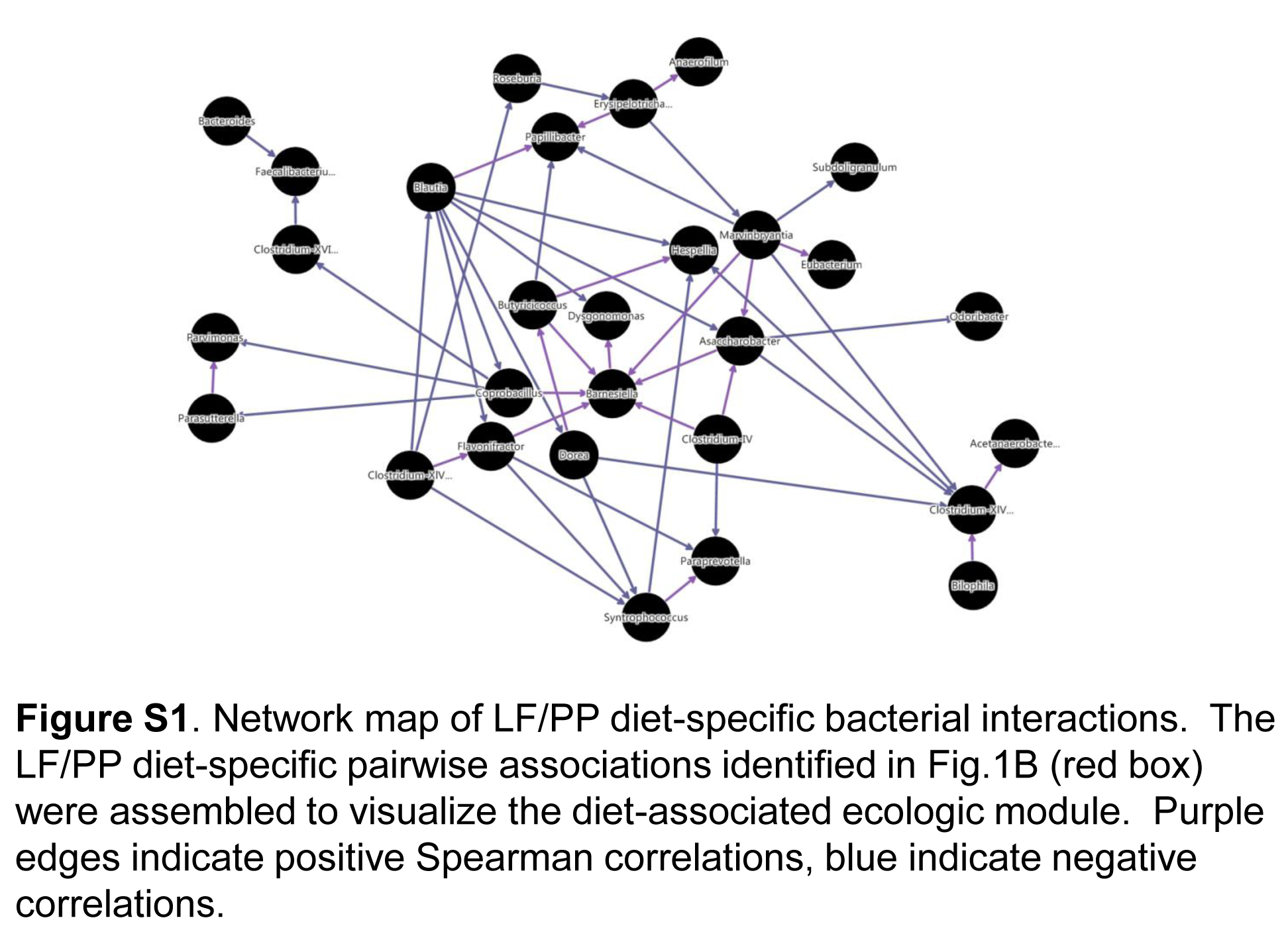

Supplement: Figure S1 — Network map of LF/PP diet-specific bacterial interactions. The LF/PP diet-specific pairwise associations identified in Fig.1B (red box) were assembled to visualize the diet-associated ecologic module. Purple edges indicate positive Spearman correlations, blue indicate negative correlations. (TIF) [file pone.0083689.s001.tif]

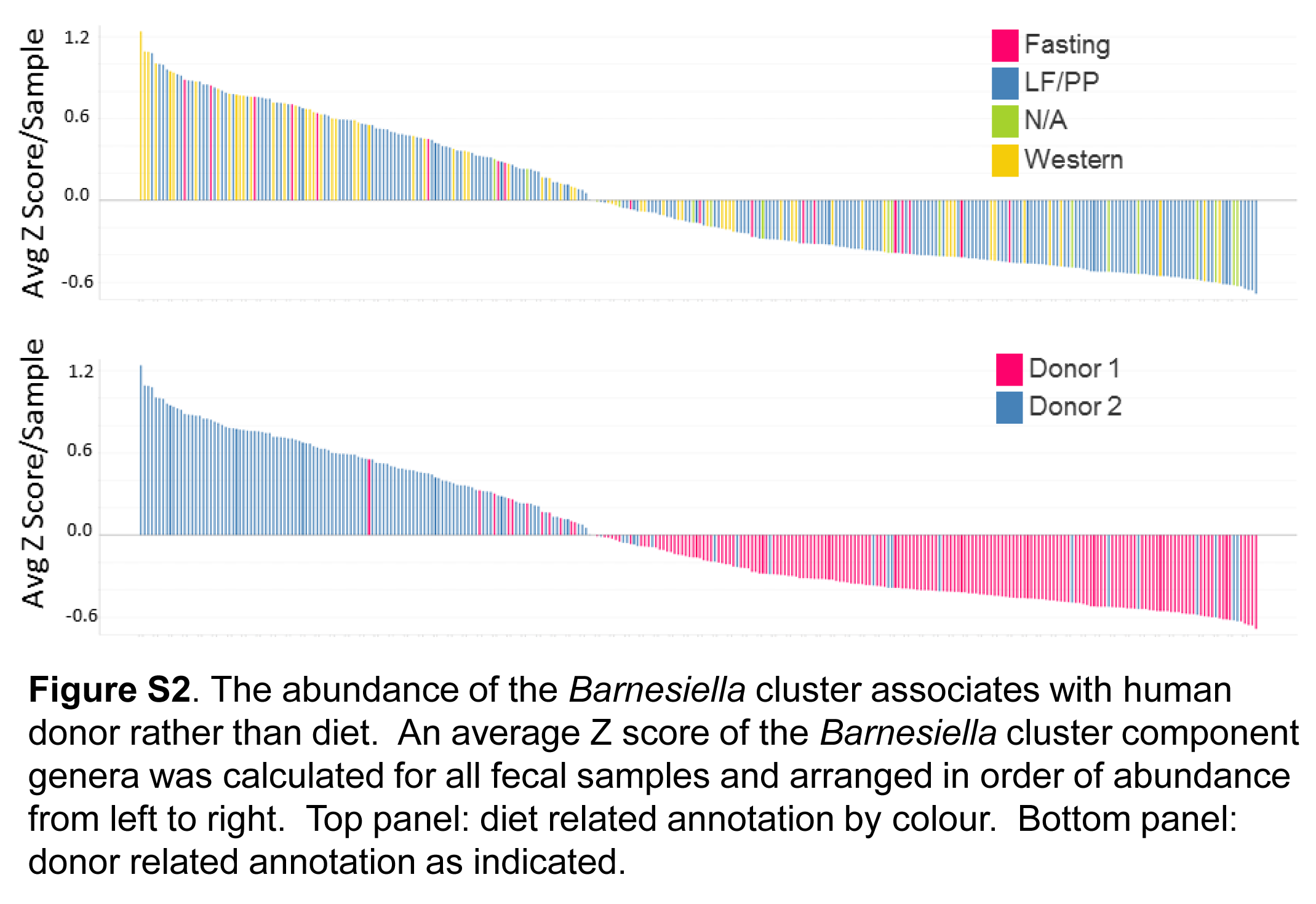

Supplement: Figure S2 — The abundance of the Barnesiella cluster associates with human donor rather than diet. An average Z score of the Barnesiella cluster component genera was calculated for all fecal samples and arranged in order of abundance from left to right. Top panel: diet related annotation by colour. Bottom panel: donor related annotation as indicated. (TIF) [file pone.0083689.s002.tif]

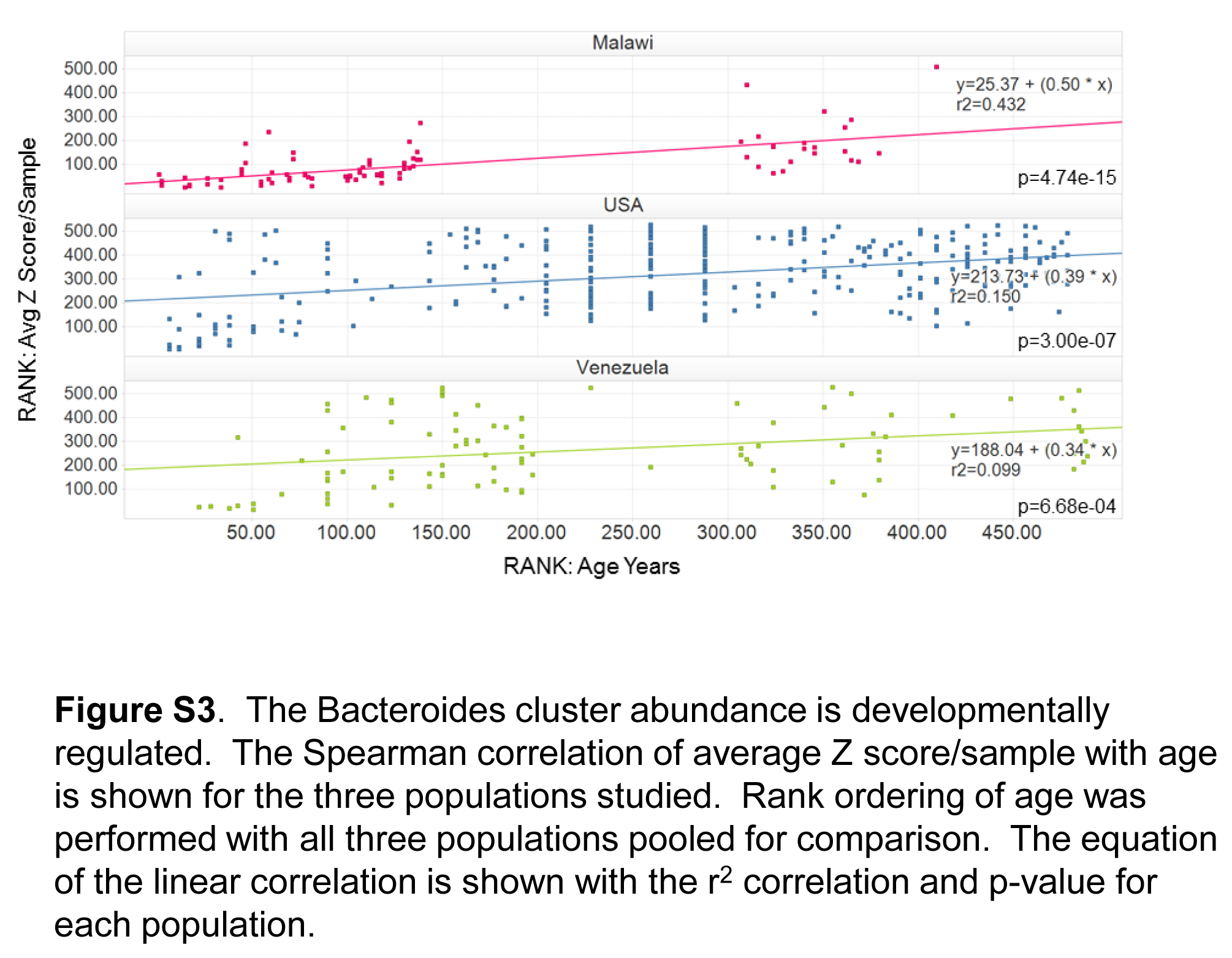

Supplement: Figure S3 — The Bacteroides cluster abundance is developmentally regulated. The Spearman correlation of average Z score/sample with age is shown for the three populations studied. Rank ordering of age was performed with all three populations pooled for comparison. The equation of the linear correlation is shown with the r2 correlation and p-value for each population. (TIF) [file pone.0083689.s003.tif]
